# A Rare Axillary Lymph Node Metastasis on ^18^F-FDG PET/CT for Staging in a Patient with Common Bile Duct Cancer

**DOI:** 10.3390/diagnostics13183012

**Published:** 2023-09-21

**Authors:** Yeongjoo Lee, Hye Sung Won, Kyung Jin Seo, Sae Jung Na

**Affiliations:** 1 Department of Radiology, Uijeongbu St. Mary’s Hospital, College of Medicine, The Catholic University of Korea, Seoul 06591, Republic of Korea; soap2222@msn.com; 2 Division of Medical Oncology, Department of Internal Medicine, Uijeongbu St. Mary’s Hospital, College of Medicine, The Catholic University of Korea, Seoul 06591, Republic of Korea; 3 Department of Hospital Pathology, Uijeongbu St. Mary’s Hospital, College of Medicine, The Catholic University of Korea, Seoul 06591, Republic of Korea

**Keywords:** common bile duct cancer, axillary lymph node, metastasis, ^18^F-FDG PET

## Abstract

Distant metastasis of cholangiocarcinoma is most commonly diagnosed in the liver; however, it can also be found in the lungs, distant lymph nodes, bones, and brain. Distant lymph node metastasis outside the abdominal region without concurrent abdominal metastasis is exceedingly rare in extrahepatic cholangiocarcinoma. Herein, we present interesting ^18^F-FDG PET/CT images of a 49-year-old male patient with common bile duct cancer. In this case, the patient, who was scheduled for surgery, unexpectedly showed axillary lymph node metastasis on a preoperative ^18^F-FDG PET scan, which was subsequently confirmed via histological examination. Although such cases are exceptionally rare, this accurate diagnosis prompted a modification of the treatment plan, leading to a positive therapeutic response.

**Figure 1:**
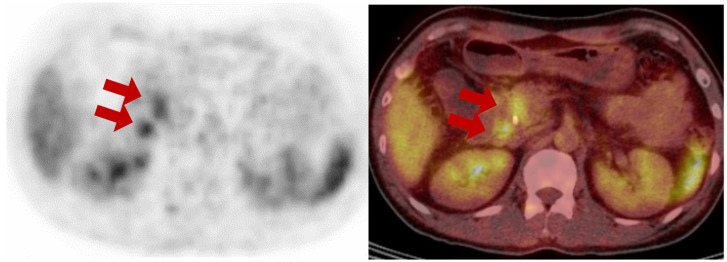
A 49-year-old male presented with abdominal discomfort and underwent computed tomography (CT) at a local clinic which revealed dilatation of the common bile duct (CBD) and intrahepatic ducts (IHD). The patient was referred to a specialized center for further evaluation to exclude CBD cancer. The laboratory results revealed a normal white blood cell count of 7550/µL, a typical hemoglobin level of 13.0 g/dL, and a platelet count within the expected range of 239 × 103/µL. Kidney function appeared normal, with a blood urea nitrogen (BUN) level of 11.6 mg/dl and a creatinine level of 0.7 mg/dL. However, there were significant abnormalities in liver function, including elevated levels of aspartate aminotransferase (AST) at 175 U/L, alanine aminotransferase (ALT) at 295 U/L, and alkaline phosphatase (428 U/L). The total bilirubin level was notably high at 11.54 mg/dL, with direct bilirubin level of 10.45 mg/dL. Furthermore, gamma-glutamyl transferase levels were substantially elevated (1255 U/L). In addition, an elevated C-reactive protein level (1.31 mg/dL) was also observed. His body mass index (BMI) was 23.9, and he had no history of diabetes, hypertension, or fatty liver disease. Endoscopic retrograde cholangiopancreatography (ERCP), biopsy, and endoscopic nasobiliary drainage (ENBD) insertion were performed to confirm the diagnosis. The pathological results of the CBD biopsy confirmed the presence of a moderately differentiated adenocarcinoma. To determine the stage of cancer, MRI of the liver and a ^18^F-FDG PET/CT scan were conducted. The ^18^F-FDG PET/CT scan showed focal uptake in the mid-CBD, where a biopsy was performed, indicating CBD cancer. The axial PET and fusion images from ^18^F-FDG PET/CT show focal uptake (red arrows) in the common bile duct with a drainage catheter and the posterior duodenopancreatic area, suggesting biopsy-proven primary cancer and a regional metastatic lymph node, respectively.

**Figure 2:**
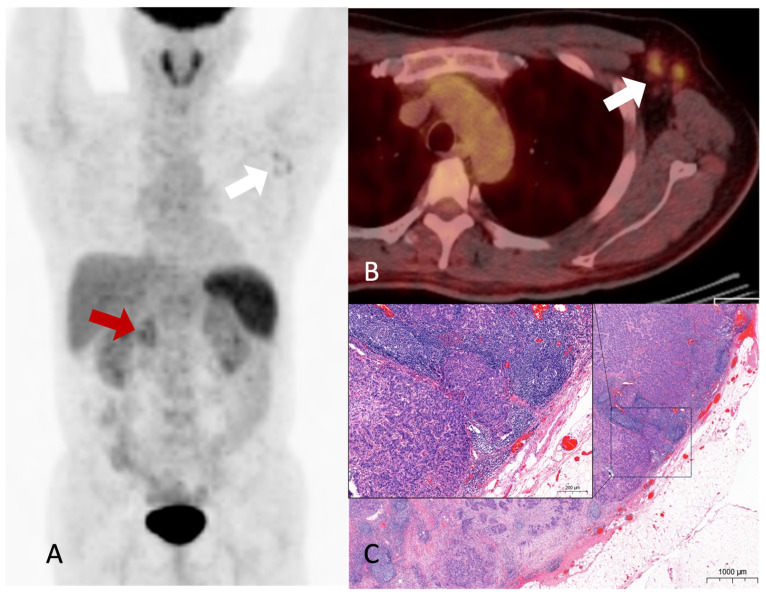
Hypermetabolic nodular activities are noted in the common bile duct and duodenopancreatic area (**A**, red arrow), and left axillary area (**A**, white arrow) on the maximal-intensity projection image of the ^18^F-FDG PET/CT. Hypermetabolic enlarged lymph nodes are noted in the fusion axial image (**B**, white arrow). No abnormal hypermetabolic activity was observed in the liver or lungs. Although diffuse uptake was observed in the spleen, no specific abnormalities were detected on contrast-enhanced abdominal CT and MRI imaging. This finding was presumed to be related to the patient’s underlying anemia, and it required further confirmation during follow-up. Furthermore, there was no evidence of splenic metastasis during follow-up. Moderately hypermetabolic lymph nodes in the left axillary area were difficult to exclude metastasis completely. An excisional biopsy of the axillary lymph nodes was performed because the treatment was changed from surgery to systemic therapy based on the presence or absence of distant metastases. Histological reports revealed the presence of adenocarcinoma, which was consistent with metastasis from CBD cancer. An H&E-stained slide of the axillary lymph node showing metastatic carcinoma (**C**). Accordingly, the patient’s treatment plan was modified; further, gemcitabine, cisplatin, and paclitaxel regimens were administered, resulting in a partial response with good performance. Subsequently, a surgical procedure (a pylorus-preserving pancreaticoduodenectomy) was performed. The patient survived for 2 years and 1 month after the diagnosis. Although there have been recurrences after surgery, the patient is currently maintaining a stable status through palliative chemotherapy. Metastasis is a hallmark of cancer and the leading cause of cancer-related death. Although many advances have been made in understanding metastasis, it is not yet fully understood [1]. The liver is the most common (11.9–23.2%) distant metastatic site of extrahepatic bile duct cancer, and metastases are known to be diagnosed in the lung, distant lymph nodes, bone, or brain [2]. Distant metastasis to the axillary lymph nodes from the primary tumor in the abdomen is rare. In particular, it is rarely discovered at the time of diagnosis except in cases with extensive lymph node metastasis to the abdomen or distant metastasis to other organs [3]. The case of recurrence due to axillary metastasis during post-operative follow-up is also very rare, thereby warranting a case report [4]. One case has been reported in which a patient with metastases to the abdominal and axillary lymph nodes, for whom the primary cancer origin could not be identified, was diagnosed with intrahepatic cholangiocarcinoma through a postmortem examination [5]. As with any type of cancer, accurate staging is essential for the selection of an appropriate treatment and a favorable prognosis. If distant metastases are not recognized and local treatment is performed, especially in patients being considered for surgery based on suspected primary tumor and regional lymph node involvement, appropriate treatment is delayed, and the patient’s prognosis is inevitably poor. In the current patient, surgical treatment was feasible only when an abdominal CT scan and MRI were initially performed. However, following the ^18^F-FDG PET/CT scan, axillary lymph node metastasis was suspected, leading to the confirmation of distant metastasis through tissue examination. As a result, the treatment approach shifted to systemic therapy, and the patient responded to the treatment. ^18^F-FDG PET/CT is known to effectively diagnose distant metastases in various cancers [6], and many reported cases have highlighted its ability to detect metastases in unexpected locations [7,8,9]. A previous study showed that for approximately 15% of patients with suspected and potentially operable cholangiocarcinoma, treatment altered with ^18^F-FDG PET/CT scans [10]. However, there have been no reported cases of axillary lymph node metastasis without other distant metastases identified during the staging process in patients with CBD cancer who are being considered for surgery. Herein, we emphasize the importance of determining treatment through an accurate diagnosis before surgery, elucidating a case in which unexpected distant axillary lymph node metastasis was discovered following ^18^F-FDG PET/CT in a patient with extrahepatic cholangiocarcinoma who was scheduled for surgery.

## Data Availability

The data presented in this study are available upon request from the corresponding author.
